# A Novel Radiomics-Integrated Panel for Preoperative Stratification of Pancreatic Neuroendocrine Tumors (PNETs)

**DOI:** 10.3390/cancers18101663

**Published:** 2026-05-21

**Authors:** Abdallah Attia, Jihun Hamm, Mahmoud A. AbdAlnaeem, Zhengming Ding, Michael O’Rorke, Joseph Dillon, Mary Maluccio, Nicholas Skill, Kristen Limbach

**Affiliations:** 1 Department of Surgery, Tulane University School of Medicine, New Orleans, LA 70112, USA; aattia@tulane.edu (A.A.); mabdalnaeem@tulane.edu (M.A.A.); 2 Department of Computer Science, Tulane University School of Science and Engineering, New Orleans, LA 70118, USA; jhamm3@tulane.edu (J.H.); zding1@tulane.edu (Z.D.); 3 Department of Surgery, University of Iowa Carver College of Medicine, Iowa City, IA 52242, USA; michael-ororke@uiowa.edu (M.O.); joseph-dillon@uiowa.edu (J.D.); 4 Department of Surgery, Louisiana State University Health Sciences Center, New Orleans, LA 70112, USA; mmaluc@lsuhsc.edu (M.M.); nskill@lsuhsc.edu (N.S.)

**Keywords:** pancreatic neuroendocrine tumors, CT radiomics, Δ-radiomics, ComBat harmonization, preoperative risk stratification, calibration, nested cross-validation

## Abstract

**Background.** Preoperative risk stratification of pancreatic neuroendocrine tumors (PNETs) is constrained by the unavailability of histologic grade before resection. We hypothesized that a panel of biologically informed CT-radiomic signatures, combined with patient-level Δ-radiomics referenced to the contralateral pancreas, would support preoperative discrimination of progression and grade in a two-center pilot cohort. **Methods.** Forty-four patients with histologically confirmed PNET who underwent contrast-enhanced preoperative CT and surgical resection at two academic centers were analyzed. Lesion and contralateral non-tumor-bearing pancreatic parenchyma regions of interest were revised in 3D Slicer by a board-certified pancreatic surgeon and verified intraoperatively against surgical pathology. PyRadiomics v3.0 features were extracted with IBSI-concordant settings. Parametric ComBat batch correction was applied across the two centers (biological-covariate balance verified beforehand), and Δ-radiomic features (lesion combat–pancreas combat) were computed for the 106 intensity/texture primitives. We constructed a panel of biology-informed hybrid signatures partitioned into a preoperative lesion-only family (Family A; seven signatures) and a preoperative Δ-radiomic family (Family B; three signatures). Candidate features were filtered through correlation clustering, baseline-adjusted likelihood-ratio testing with Benjamini–Hochberg FDR control, and 100-bootstrap stability selection. Three predictor blocks were compared per target with three classifiers each (Logistic Regression, Random Forest, Gradient Boosting): M0 (five-variable clinical baseline), MA (M0 + Family A), and MB (M0 + Family B). Discrimination was reported as AUC with bootstrap 95% CI; calibration was assessed using the Brier score and TRIPOD-recommended calibration intercept and slope; and cross-center generalization was evaluated with leave-one-center-out (LOCO) cross-validation. Univariable Cox regression with bootstrap and permutation inference was used for progression-free survival (PFS). **Results.** The cohort had 16 progression events and eight deaths (median follow-up was 38 months, IQR 14–59). Prespecified clinical–radiomic and Δ-radiomic signatures were associated with progression-free survival, including B2 = ΔBusyness × Ki-67 (HR 0.38, 95% CI 0.19–0.76, *p* = 0.006). For progression prediction, the Δ-radiomic model achieved the strongest discrimination, with a nested cross-validation AUC of 0.85 and leave-one-center-out AUC of 0.87. For higher-grade disease, radiomic models also demonstrated high discrimination, with AUCs up to 0.93. **Conclusions.** Radiomics-derived shape and texture features, especially when combined with clinical markers, may noninvasively identify aggressive PNET phenotypes and support preoperative risk stratification. Prospective validation in larger multicenter cohorts is warranted.

## Simple Summary

Pancreatic neuroendocrine tumors (PNETs) are clinically heterogeneous neoplasms whose preoperative risk stratification is limited by the unavailability of histologic grade before surgery. We tested whether quantitative features extracted from routine preoperative CT can support preoperative risk stratification in a two-center retrospective cohort of 44 patients. We constructed a small panel of biologically informed hybrid signatures combining lesion radiomic primitives with clinical variables. We additionally constructed Δ-radiomic features that use each patient’s contralateral pancreas as an internal control. After ComBat batch correction across the two contributing centers, the Δ-radiomic signature B2 (ΔBusyness × Ki-67) was the most consistent progression-associated signature on univariable Cox regression, and a parsimonious classifier built on Family B Δ-signatures exceeded the clinical baseline for progression discrimination while maintaining acceptable calibration. The findings support radiomics as a candidate adjunct to preoperative risk assessment in PNETs and motivate prospective external validation.

## 1. Introduction

Pancreatic neuroendocrine tumors (PNETs) are a heterogeneous group of neoplasms that have been increasing in incidence over recent decades [1,2,3]. The clinical behavior of these neoplasms can vary widely from indolent to highly aggressive, and prognosis is primarily determined by differentiation, histologic grade, and extent of metastasis [1,2,3,4,5]. Given this variation, determination of prognostic factors at diagnosis is critical for creation of an appropriate treatment plan, as operative resection is a cornerstone of curative treatment in lower-grade disease but may be inappropriate for high-grade disease [5]. Furthermore, accurate preoperative risk stratification is crucial for optimizing the surgical approach, as it may affect the extent of lymph-node dissection and/or consideration of neoadjuvant therapy [6,7].

National Comprehensive Cancer Network (NCCN) guidelines recommend acquisition of conventional imaging for initial workup of a newly diagnosed PNET, including a multiphasic abdomen-and-pelvis computed tomography (CT) scan or magnetic resonance imaging (MRI), with the option of adding functional imaging such as a somatostatin-receptor PET/CT in individual cases as appropriate [8]. While such imaging may allow for the technical aspects of preoperative planning, CT and MRI have poor lymph-node sensitivity and give little to no insight into grade. Therefore, imaging alone does not allow for accurate prognostication. Rather, this requires histopathologic grading based on Ki-67 proliferation index and mitotic rate, but these markers are only available after tissue acquisition. In the preoperative setting, tissue is typically obtained using endoscopic ultrasound and fine-needle biopsy. Unfortunately, Ki-67 often cannot be reliably determined from fine-needle biopsy alone [9,10,11], and a need exists for a feasible method of obtaining accurate, reliable prognostic information at the time of diagnosis.

Radiomics, the high-throughput extraction of quantitative features from medical images, offers the potential for noninvasive tumor characterization and outcome prediction [12,13]. By capturing subtle patterns in tumor texture, shape, and intensity distributions that may not be apparent to visual inspection, radiomics can provide information to conventional imaging assessment [14]. Two broad families of machine-learning models have been applied to oncologic imaging tasks, including in PNETs: end-to-end deep learning (typically convolutional neural networks, CNNs, including 2D/3D CNNs and U-Net-style architectures) and classical supervised learning trained on a fixed, pre-extracted radiomic feature set [15,16,17,18,19,20,21]. Deep models can in principle learn task-specific imaging representations directly from voxel data, but their parameter counts are typically several orders of magnitude larger than the number of patients in PNET cohorts, which is unfavorable at events-per-variable budgets typical of this disease and produces models that are difficult to interpret biologically. The role of such radiomic features in PNET prognostication remains incompletely characterized, and the question remains whether they can support robust preoperative risk stratification in this population. The aim of this study is to develop and validate radiomics-based models for PNET characterization and outcome prediction, identifying imaging characteristics that correlate with established clinical prognostic factors.

## 2. Materials and Methods

### 2.1. Cohort and Clinical Data

**Figure 1:**
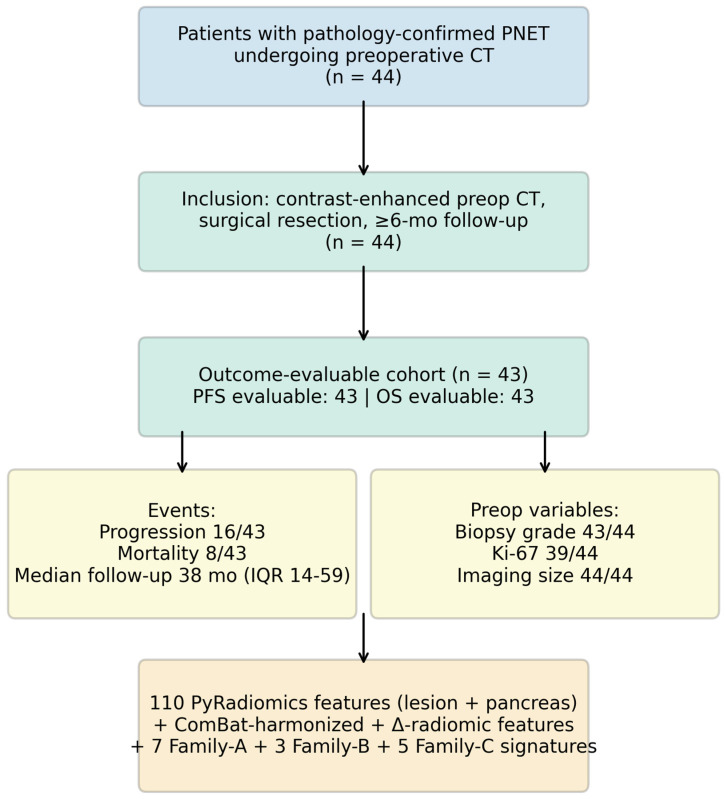
Cohort flow and analytic dataset. Inclusion required histologically confirmed PNET, contrast-enhanced preoperative CT of diagnostic quality, surgical resection, and ≥6-month follow-up. Per-variable completeness, event counts, and the final feature/signature inventory are shown in the bottom row.

A retrospective review was conducted of patients with pathologically confirmed PNET who underwent contrast-enhanced preoperative CT and surgical resection at two academic centers between 2015 and 2025. Inclusion required histopathologic confirmation of PNET, a contrast-enhanced preoperative CT of diagnostic quality, a minimum 6-month clinical follow-up, and availability of biopsy-derived grade and Ki-67. Clinical variables abstracted from the medical record included age, sex, biopsy and surgical tumor grade (WHO 2022 G1/G2/G3), biopsy Ki-67 index (%), mitotic rate (per 2 mm^2^), preoperative imaging tumor size (cm), surgical specimen tumor size (descriptive only), functional/hormonal status, diagnostic-imaging lymph-node count, surgical lymph-node count, number of metastatic organs at diagnosis, perineural and lymphovascular invasion, and progression and mortality status with dates [22]. The study was approved by the Institutional Review Boards of both participating institutions with waiver of informed consent for retrospective analysis, as shown in Figure 1.

### 2.2. CT Acquisition, Segmentation, and Radiomic Feature Extraction

All scans were contrast-enhanced multidetector CT in the portal-venous phase. Lesion and contralateral non-tumor-bearing pancreatic parenchyma regions of interest (ROIs) were revised slice-by-slice in 3D Slicer by a board-certified pancreatic surgeon with subspecialty PNET expertise, providing direct anatomic correlation with the resection specimen and pathology. Radiomic features were extracted with PyRadiomics v3.0 [23] using settings concordant with the Image Biomarker Standardization Initiative [24]: isotropic resampling to 1 mm × 1 mm × 1 mm with B-spline interpolation, intensity resegmentation to the [−150, 240] HU range, fixed bin width of 25 HU, and computation across the seven feature classes (first-order, shape, GLCM, GLRLM, GLSZM, GLDM, NGTDM) under the original image-type filter, yielding 110 lesion-derived features per patient. To address shape-feature collinearity, the 14 lesion shape descriptors were summarized by principal-component analysis, retaining the first three components (cumulative variance explained 93.1%). The same extraction was applied to the contralateral pancreas ROI.

#### 2.2.1. ComBat Batch Harmonization

Routine clinical CT acquisitions across the two contributing centers and multiple scanner generations introduced spatial and intensity-domain heterogeneity. The 1 mm × 1 mm × 1 mm B-spline resampling above standardizes spatial geometry; to mitigate the residual intensity-domain batch effect we applied parametric ComBat batch correction to all lesions + pancreas radiomic features simultaneously, with center as the batch variable. Before applying ComBat we verified the balance of biological covariates across the two centers using the Mann–Whitney U test [25,26].

#### 2.2.2. Δ-Radiomics

Each patient’s contralateral pancreas served as a per-patient internal control intended to absorb residual scanner bias, contrast-timing offset, and body habitus that ComBat at the center level cannot capture. For every PyRadiomics intensity/texture primitive *X* with both a lesion combat and a pancreas combat value we computed Δ*X_i_* = *X*_i_^lesion^^,^^combat^ − *X*_i_^pancreas,combat^, producing 106 Δ-features (shape primitives are dominated by lesion-vs-pancreas volume differences and are excluded). The Δ-pool is the basis of Family B signatures.

### 2.3. Hybrid Signature Panel

A panel of biology-informed hybrid signatures was constructed a priori and partitioned by family. Family A (7 preoperative signatures, lesion-only) uses variables knowable before surgery and spans six biologically distinct PNET aggressiveness axes (proliferation × histogram heterogeneity, morphology, functional/grade, metastatic burden, vascular enhancement, spatial intratumoral heterogeneity), plus one explicit three-way interaction (A5 = A1 × A2). Every Family A signature contains at least one clinical multiplicand. Family B (3 preoperative signatures, Δ-radiomic) replaces the lesion-only radiomic primitive of selected Family A signatures with its corresponding Δ-feature: B1 = ΔEntropy × Ki-67, B2 = ΔBusyness × Ki-67, B3 = ΔMedianHU × Grade. The hypothesis is that subtracting the per-patient internal-control reference will increase the predictive value of the same hybrid interaction by removing scanner/body-habitus/contrast-timing variation shared between the tumor ROI and the contralateral pancreas Table 1.

**Table 1:** Preoperative biology-informed hybrid signatures.

ID	Name	Formula	Biological Axis
A1	Proliferation × histogram heterogeneity	Entropy × Ki-67(fraction)	Proliferation × histogram heterogeneity
A2	Morphologic complexity	(Surface/Volume) × (1 − Sphericity)	Morphology
A3	Functional-morphologic-grade	(1 − Sphericity) × Functional × Grade(biopsy)	Functional × morphology × grade
A4	Metastatic burden	log(Energy + 1) × Grade(biopsy) × (1 + N metastatic organs)	Intensity × differentiation × imaging-staged spread
A5	Proliferation × complexity (3-way)	A1 × A2	Proliferation × heterogeneity × morphology
A6	Spatial heterogeneity × proliferation	NGTDM Busyness × Ki-67(fraction)	Spatial heterogeneity × proliferation
A7	Vascular × differentiation	Median HU(lesion) × Grade(biopsy)	Portal-venous-phase intensity × differentiation
B1	Δ-Proliferation	(Entropy lesion—Entropy pancreas) × Ki-67(fraction)	Heterogeneity above pancreatic baseline × proliferation
B2	Δ-Spatial × proliferation	(Busyness lesion—Busyness pancreas) × Ki-67(fraction)	Spatial heterogeneity above baseline × proliferation
B3	Δ-Vascular × differentiation	(MedianHU lesion—MedianHU pancreas) × Grade(biopsy)	Tumor enhancement above pancreatic baseline × differentiation

Entropy = Shannon information entropy of the CT intensity histogram within the ROI; NGTDM Busyness = Neighborhood Grey-Tone Difference Matrix Busyness (voxel-to-voxel intensity changeability); Median HU = median Hounsfield-unit intensity within the ROI on portal-venous-phase CT; Surface/Volume and Sphericity are PyRadiomics shape descriptors. Ki-67 is converted to a fraction (0–1) before multiplication. Family B Δ features are computed on ComBat-harmonized values (lesion combat—pancreas combat).

### 2.4. Statistical Analysis

Analyses were performed in Python 3.12, scikit-learn v1.6, lifelines v0.30, pandas v2.0, and numpy v1.24, with statsmodels v0.14 and the combat package for batch harmonization. Continuous variables are presented as median (IQR); categorical variables as count (%). Comparisons between groups were performed using Mann–Whitney U or Kruskal–Wallis tests as appropriate. Associations between imaging and clinical continuous variables were assessed with Spearman rank correlation and controlled for multiple comparisons using the Benjamini–Hochberg false discovery rate (FDR *q* < 0.05).

Progression-free survival used the right-censored time-to-event variable, with patients at last follow-up if no progression occurred. Each signature was z-standardized within its complete-case subset and entered univariable Cox proportional-hazards regression. Hazard ratios are reported per 1-SD increase with profile-likelihood 95% CIs. Bootstrap 95% confidence intervals for the concordance index (2000 resamples) and permutation *p*-values for the concordance (1000 permutations) were computed for each signature. Median-split Kaplan–Meier curves were generated with pointwise 95% bands and log-rank tests.

Predictive modeling. We (i) used 5 preoperative clinical baseline (M0) variables to establish prognostic relevance; (ii) screened candidate radiomic features through correlation clustering at |Spearman ρ| ≥ 0.80, baseline-adjusted likelihood-ratio testing with BH-FDR < 0.10, and 100-bootstrap stability selection at ≥ 60%. Three predictor blocks were compared per target: M0 (clinical baseline); MA = M0 + Family A; and MB = M0 + Family B. For higher-grade prediction, biopsy grade was removed from M0 and any signature whose formula contains biopsy grade (A3, A4, A7, B3) was excluded from the relevant blocks to avoid label leakage.

Three classifiers were fitted per block (Logistic Regression, Random Forest, Gradient Boosting); the SelectKBest filter searched k ∈ 2, 3, 5 inside the inner CV loop. Outer 5-fold stratified cross-validation produced unbiased out-of-fold predictions; inner 5-fold cross-validation performed *k*-selection. Imputation, scaling, and selection were performed inside the cross-validation pipeline. Discrimination was reported as AUC with bootstrap 2000-resample 95% CIs. Calibration was reported as Brier score plus the TRIPOD-recommended calibration intercept and slope *b* [27] from the logistic regression of the binary outcome on logit(p_i_), with bootstrap 95% CIs. As the strongest internal proxy for external validation in a two-center cohort we additionally performed leave-one-center-out (LOCO) cross-validation per block per target, reporting the pooled out-of-fold AUC.

## 3. Results

### 3.1. Study Population

Of 44 patients with imaging data the median age was 62 years (IQR 58–68); 56.8% were male. Biopsy grade was G1 in 24 (54.5%), G2 in 15 (34.1%) and G3 in four (9.1%). The median preoperative imaging tumor size was 2.7 cm (IQR 1.5–4.6). Six patients (13.6%) had functional tumors. Sixteen patients (37.2%) experienced disease progression and eight (18.6%) died during follow-up; the median follow-up was 38 months (IQR 14–59). Baseline characteristics are summarized in Table 2.

### 3.2. Lesion-Vs-Pancreas Discrimination Validates Radiomic Phenotyping

**Figure 2:**
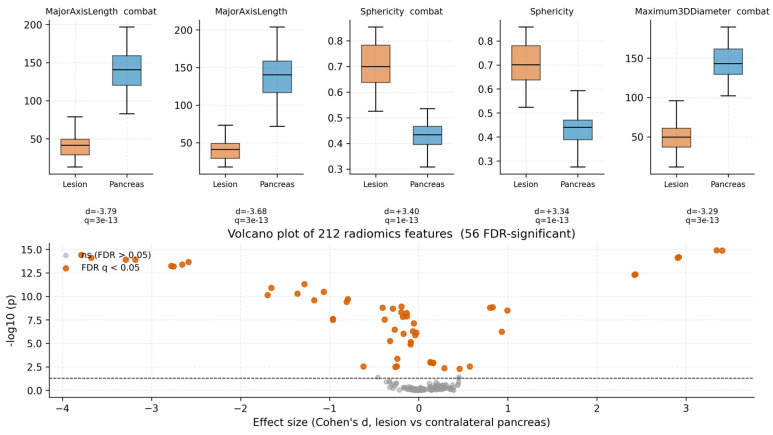
Top distinguishing radiomic features (lesion vs. contralateral pancreas). Boxplots show five representative top-ranked lesion-derived features (left = lesion ROI; right = matched pancreas ROI). *p*-values are from two-sided Mann–Whitney U tests. The volcano plot (lower right) displays effect size versus −log_10_(*p*) for all 110 features tested; the dashed line marks *p* = 0.05; highlighted points denote features surviving FDR correction (*q* < 0.05).

Among 110 lesion-derived radiomic features tested against the matched contralateral pancreas, 27 differed at FDR *q* < 0.05 (Figure 2). Shape descriptors dominated the top of the volcano plot: lesions had larger major axis length (Cohen’s *d* = −3.68, *q* = 3 × 10^−13^), maximum 3D diameter (*d* = −3.18), and reduced sphericity (*d* = +3.34) and flatness (*d* = +2.90). Texture features (GLSZM, GLCM Idmn) and first-order energy/total energy contributed an additional cluster of significant differences with smaller effect sizes. These differences confirm that the radiomic feature space distinguishes PNET from normal pancreatic parenchyma in this cohort.

### 3.3. Radiomic–Clinical Correlations Identify Reproducible Imaging Surrogates

**Figure 3:**
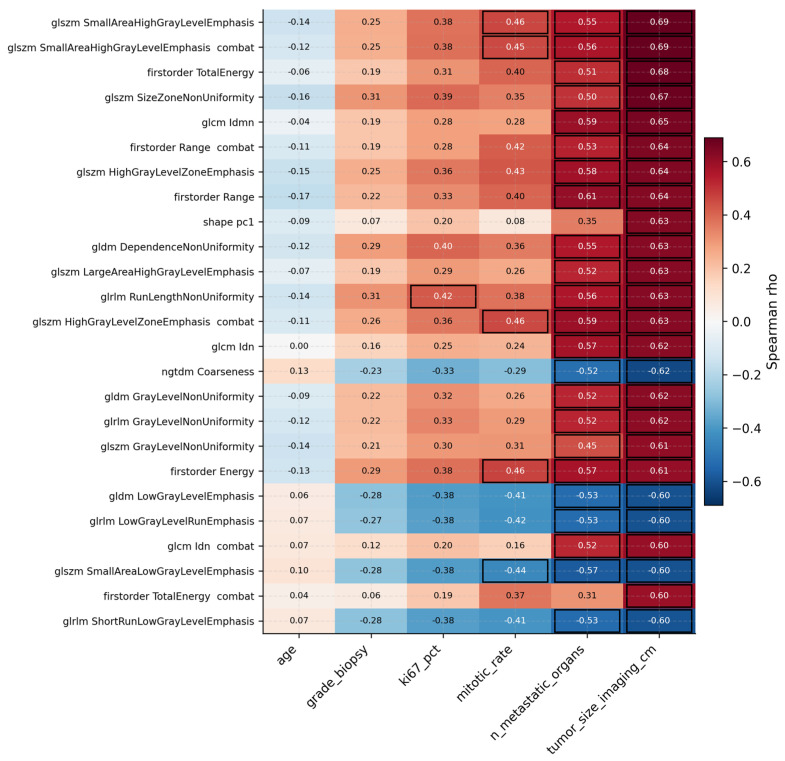
Heatmap of Spearman correlations between the top 20 lesion-derived radiomic features and preoperative clinical variables. Black-outlined cells denotes associations surviving FDR correction (*q* < 0.05).

Across the radiomic–clinical pairwise correlations, Figure 3 shows that the strongest associations were between texture/shape features and preoperative imaging tumor size: GLSZM SmallAreaHighGrayLevelEmphasis ρ = +0.69, first-order TotalEnergy ρ = +0.68, and GLSZM SizeZoneNonUniformity ρ = +0.67. The shape PC1 composite reached ρ = +0.63. Correlations with mitotic rate, Ki-67, and grade were weaker but consistent in direction, with several first-order intensity and texture features (Energy, Range, GLDM DependenceNonUniformity) showing positive trends with proliferation markers.

### 3.4. Survival Analysis

**Figure 4:**
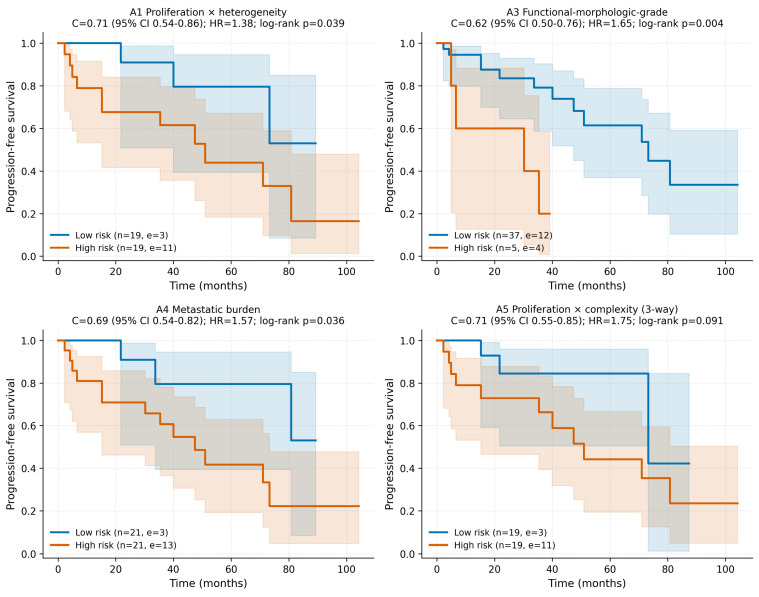
Kaplan–Meier progression-free-survival curves stratified by median split for four representative Family A preoperative signatures. Solid lines represent Kaplan–Meier estimates; shaded bands indicate pointwise 95% confidence intervals (Greenwood). Tick marks denote censored observations. Insets report hazard ratio per 1-SD increase with 95% CI, bootstrap concordance, and log-rank *p*-value. Time axis in months.

Univariable Cox results for the 10 preoperative signatures on PFS are summarized in Table 3. Five signatures showed statistically significant Cox effects: A3 functional–morphologic (HR 1.65 per SD, 95% CI 1.12–2.43, *p* = 0.012), A4 metastatic burden (HR 1.57, 1.03–2.41, *p* = 0.037), A5 proliferation × complexity (HR 1.75, 1.12–2.74, *p* = 0.014; concordance 0.71, 95% CI 0.55–0.85; permutation *p* = 0.021), A7 vascular × differentiation (HR 1.69, 1.07–2.66, *p* = 0.025), and the Δ-radiomic signature B2 = ΔBusyness × Ki-67 (HR 0.38, 95% CI 0.19–0.76, *p* = 0.006) in the protective direction. B2 was the most consistent progression-associated Δ-radiomic signature in this cohort. The Family B non-survivors B1 (ΔEntropy × Ki-67) and B3 (ΔMedianHU × Grade) were not Cox-significant. Median-split Kaplan–Meier curves with Greenwood 95% bands and log-rank *p*-values for the four highest-discriminating Family A signatures are shown in Figure 4 with time in months.

### 3.5. Predictive Modeling

**Figure 5:**
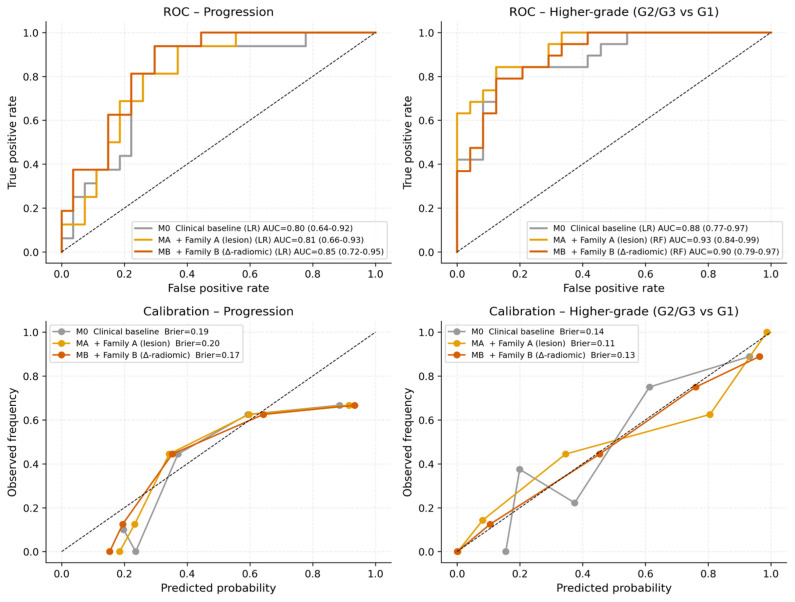
Discrimination and calibration (nested 5 × 5 CV) for the three predictor blocks (M0, MA, MB) on the two endpoints (progression, higher-grade). Top row: ROC curves with AUCs and bootstrap 95% CIs. Bottom row: calibration/reliability diagrams with Brier scores.

**Figure 6:**
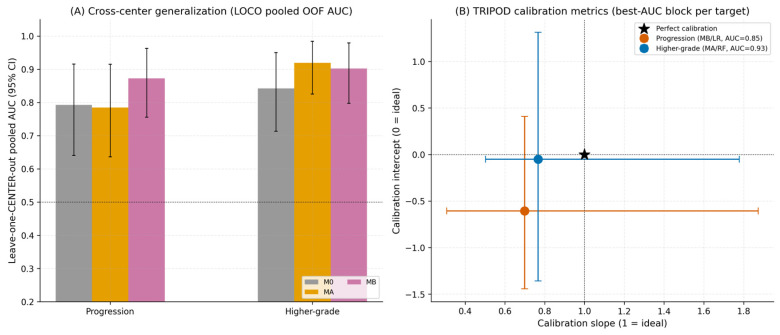
Cross-center generalization and TRIPOD calibration metrics. (**A**) Leave-one-center-out (LOCO) pooled out-of-fold AUC for the three predictor blocks (M0, MA, MB) per endpoint (progression, higher-grade). Error bars = 1000-bootstrap 95% CI on the pooled OOF predictions. (**B**) TRIPOD calibration intercept (perfect = 0) and slope (perfect = 1) with bootstrap 95% CIs for the best-AUC block per endpoint.

**Figure 7:**
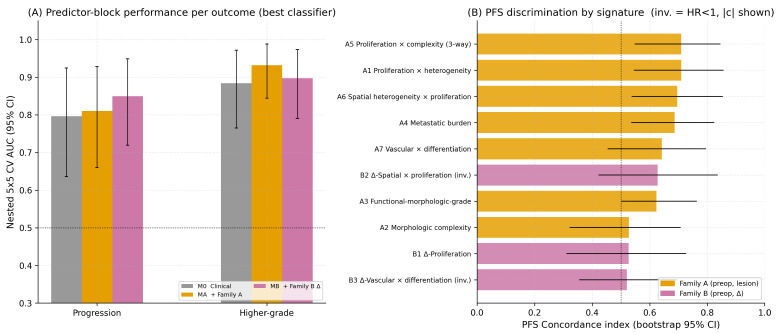
Predictive performance and signature-level survival discrimination. (**A**) Best-classifier-per-block AUC across the three predictor blocks (M0, MA, MB) per endpoint (progression, higher-grade), with bootstrap 95% CI error bars. (**B**) PFS concordance index per signature for Families A and B; for inverse-direction features (HR < 1) the discrimination equivalent |c| = max(c, 1 − c) is plotted (suffix “(inv.)”).

Candidate radiomic primitives and hybrid signatures were filtered through correlation clustering, baseline-adjusted likelihood-ratio testing with Benjamini–Hochberg false discovery rate (BH-FDR) control, and 100-bootstrap stability selection. Nested 5 × 5 cross-validation performance for the three predictor blocks is summarized in Table 4 and Figure 5, with cross-center generalization (LOCO-pooled AUC) and TRIPOD-aligned calibration analyses summarized in Figure 6. Lower Brier scores indicate better overall probabilistic prediction accuracy. For progression prediction, the Δ-radiomics block MB produced the best discrimination (AUC 0.85, 95% CI 0.72–0.95), the lowest Brier score (0.17), and the strongest LOCO-pooled cross-center AUC (0.87). For higher-grade prediction, the Family A block MA achieved the highest AUC (0.93, 95% CI 0.84–0.99) and lowest Brier score (0.11), with MB performing similarly (AUC 0.90, Brier 0.13, LOCO 0.90). The clinical baseline model alone achieved AUC 0.88 for higher-grade prediction, likely reflecting the inclusion of biopsy Ki-67 within the M0 baseline variables. Figure 7.

The observed performance split was mechanistically interpretable. Higher-grade prediction appeared to be better captured by lesion-centered Family A multiplicative biologic–radiomic interactions, whereas progression prediction benefited more from the per-patient internal-control framework used in the Δ-radiomics Family B constructs. Calibration slopes below 1 suggest residual optimism despite nested resampling and cross-center validation procedures. Performance estimates should therefore be interpreted cautiously given the modest cohort size, although nested resampling and LOCO validation were incorporated to reduce overfitting and center-specific bias.

## 4. Discussion

This study demonstrates that quantitative radiomic features extracted from preoperative CT can identify imaging characteristics associated with PNET prognosis and preoperative risk stratification. The key findings of this investigation include strong correlations between shape-based radiomic features and tumor size after multiple-comparison correction, associations between first-order intensity and texture features with proliferation markers, and moderate-to-good predictive performance for progression and higher-grade disease using nested cross-validation, calibration analysis, and leave-one-center-out cross-center validation.

The observed associations have strong biological rationale. In other malignancies, enhancement level and associated fibrosis has been associated with prognosis [28], and imaging characteristics such as mass formation have been associated with E-cadherin expression and metastatic potential in gastric adenocarcinoma [29]. Although specific associations of genomic mutations with mass formation or shape have not been noted in PNETs specifically, it follows that there may be biologic changes that produce differences in radiomics features that may then subsequently explain variability in prognosis. Regardless of the underlying biologic changes, shape correlations with tumor size validate the accuracy of radiomics measurements and suggest that imaging-derived morphometric features can serve as reliable surrogates for pathologic measurements. First-order intensity correlations with proliferation indices likely reflect underlying tissue heterogeneity, with higher Ki-67 tumors showing increased cellular density and metabolic activity that manifests as altered CT attenuation patterns. In addition, GLSZM feature importance across models suggests that zone-based texture analysis captures clinically relevant heterogeneity. Large-area emphasis and low-gray-level-zone emphasis may reflect necrosis, cystic change, or variable enhancement patterns associated with tumor aggressiveness. The discrimination achieved is broadly consistent with prior CT-based PNET radiomics studies. In a 138-patient single-center cohort, Liang et al. reported a combined CT-radiomics + clinical nomogram for preoperative grade prediction with AUC 0.97 in the development set and 0.90 in the internal validation set [30], and Bian et al. derived a CT radiomics score that distinguished G1 from G2 nonfunctioning PNETs with AUC ≈ 0.86 in 137 patients [31]. More recently, Javed et al. built a CT-derived radiomics signature for nonfunctional PNET grade [32], and Ye et al. reported an interpretable radiomics model for pathologic grade [33]; functional-imaging radiomics has been pursued in parallel by Mapelli et al. (preoperative ^68^Ga-DOTATOC PET radiomics for lymph-node assessment [34]) and Bevilacqua et al. (^68^Ga-DOTANOC PET/CT for tumor grade [35]). Deep-learning approaches such as Song et al. for recurrence on preoperative CT [19] and Klimov et al. for metastasis prediction [20] have shown promise but typically require substantially larger cohorts and are less directly interpretable than hybrid signatures. In this context, our AUCs of 0.90–0.93 (Brier 0.11–0.13) and progression AUC of 0.85 (Brier 0.17, LOCO 0.87) for the small biology-informed hybrid panel are consistent with published PNET-radiomics estimates while requiring only routinely available preoperative inputs. The use of nested cross-validation, bootstrap confidence intervals, permutation testing, leave-one-center-out validation, and TRIPOD-recommended calibration intercept and slope [27,36] provides more realistic performance estimates than studies that rely on headline AUC alone.

The Δ-radiomic family represents a methodological departure from the standard delta-radiomics paradigm. In its established usage, delta-radiomics quantifies temporal change in a feature between two acquisitions of the same lesion—typically pre- versus mid- or post-treatment imaging—and has been shown to improve outcome prediction in non-small-cell lung cancer treated with stereotactic body radiotherapy [37] and in rectal cancer treated with MR-guided chemoradiotherapy [38], with consistent signal across tumor sites in the systematic review by Nardone et al. [39]. Our formulation is conceptually distinct: rather than subtracting a temporal baseline, we subtract a spatial per-patient baseline computed from the contralateral, non-tumor-bearing pancreatic parenchyma after ComBat harmonization. The mathematical motivation is a simple variance decomposition. For any radiomic feature f, the measured lesion value can be written approximately as f-lesion ≈ f-tumor biology + f-patient + f-scanner + ε, where the patient and scanner terms are shared between the lesion and any other ROI from the same patient and same acquisition. The contralateral pancreas inherits the same f-patient + f-scanner contributions but contributes no tumor signal, so subtracting it cancels these shared nuisance components and isolates the tumor-specific term. This is the same logic underlying ΔΔCt normalization in qPCR and reference-region normalization in PET imaging (e.g., SUV ratios to a reference organ), and it is complementary to population-level ComBat harmonization, because ComBat removes the between-center batch effect while the Δ formulation additionally removes the within-patient nuisance signal that ComBat is not designed to address. Busyness is an NGTDM measure of voxel-to-voxel intensity changeability and rises with intratumoral spatial heterogeneity; high lesion busyness combined with high pancreatic-baseline busyness would inflate the lesion-only formulation without reflecting tumor biology, whereas the Δ form down-weights such cases. We treat this directionality as hypothesis-driven. External replication in an independent cohort that also acquires a contralateral pancreas internal reference is required.

The moderate associations between first-order intensity features and Ki-67 (r ~ 0.38) align with previous findings and emphasize the challenge of predicting microscopic features from macroscopic imaging [40,41]; these findings suggest that radiomic features may be of benefit in preoperative risk stratification and prognostication.

Specifically, the strong correlations of shape-size with prognosis and the moderate associations of diverse radiomics features with proliferation markers suggest radiomics could complement conventional imaging assessment, particularly in cases where biopsy is not feasible or representative. However, the wide confidence intervals observed indicate substantial uncertainty that limits immediate clinical application and will require validation in a larger clinical cohort. Regardless, the prognostic value of combined imaging-clinical features (proliferation score) suggests that integrated models may outperform either modality alone.

This study is limited by a modest cohort size. The events-per-variable ratio of approximately 1.0 for survival analysis constrains multivariable modeling, and the moderate progression rate (36%, 16 events) and small number of higher-grade events (G2/G3) limit the precision of effect estimates and may not reflect the full spectrum of PNET behavior. Manual single-rater segmentation by an experienced pancreatic surgeon, with intraoperative correlation against the resection specimen and pathology, anchors the lesion contours to a direct anatomic correlate, but inter-rater reproducibility was not formally quantified in this cohort. Finally, despite the use of nested CV, the high-dimensional feature space relative to sample size means residual overfitting cannot be excluded; bootstrap CIs, permutation testing, and LOCO-CV were used to quantify the resulting uncertainty. The associations identified despite the small sample size are nonetheless biologically coherent and underscore the potential clinical utility of radiomics-integrated panels.

## 5. Conclusions

This study demonstrates that quantitative CT imaging characteristics can noninvasively support preoperative PNET risk stratification, with discrimination for progression and higher-grade disease comparable to or exceeding a variable clinical baseline. Strong correlations between shape features and tumor size validate the underlying radiomics measurements. A prespecified panel of biologically informed hybrid signatures combining radiomic primitives with clinical biomarkers provided the most consistent progression-associated signals. Future work should focus on external prospective validation, larger sample sizes, multi-rater segmentation reproducibility, and integration with additional preoperative clinical variables to develop clinically actionable prediction models for PNET management.

## Figures and Tables

**Table 2:** Patient characteristics.

Characteristic	Value
Age at diagnosis, years, median (IQR)	62 (58–68)
Sex, *n* (%) male/female	25 (56.8)/19 (43.2)
Biopsy grade G1/G2/G3, *n* (%)	24 (54.5)/15 (34.1)/4 (9.1)
Biopsy Ki-67 (%), median (IQR)	4.0 (1.5–11.0)
Imaging tumor size (cm), median (IQR)	2.7 (1.5–4.6)
Functional tumor, *n* (%)	6 (13.6)
≥1 metastatic organ at diagnosis, *n* (%)	22 (50.0)
Progression, *n* (%)	16/43 (37.2)
Mortality, *n* (%)	8/43 (18.6)
Follow-up, months, median (IQR)	38 (14–59)

**Table 3:** Univariable Cox regression for PFS by signature.

Signature	n	Events	HR per SD (95% CI)	*p* -Value
A1 Proliferation × heterogeneity	38	14	1.38 (0.90–2.13)	0.139
A2 Morphologic complexity	43	16	0.96 (0.60–1.52)	0.862
A3 Functional–morphologic grade	42	16	1.65 (1.12–2.43)	0.012
A4 Metastatic burden	42	16	1.57 (1.03–2.41)	0.037
A5 Proliferation × complexity (3-way)	38	14	1.75 (1.12–2.74)	0.014
A6 Spatial heterogeneity × proliferation	38	14	0.76 (0.40–1.46)	0.411
A7 Vascular × differentiation	42	16	1.69 (1.07–2.66)	0.025
B1 Δ-Proliferation	38	14	1.05 (0.69–1.58)	0.832
B2 Δ-Spatial × proliferation	38	14	0.38 (0.19–0.76)	0.006
B3 Δ-Vascular × differentiation	42	16	0.73 (0.49–1.08)	0.116

**Table 4:** Nested 5 × 5-CV prediction performance (best classifier per block).

Block	Progression AUC (95% CI)	Brier	Calib. Slope	LOCO AUC	Higher-Grade AUC (95% CI)	Brier	Calib. Slope	LOCO AUC
M0 (clinical baseline)	0.80 (0.64–0.92)	0.19	0.67	0.79	0.88 (0.77–0.97)	0.14	1.24	0.84
MA (+Family A)	0.81 (0.66–0.93)	0.20	0.54	0.78	0.93 (0.84–0.99)	0.11	0.77	0.92
MB (+Family B Δ)	0.85 (0.72–0.95)	0.17	0.70	0.87	0.90 (0.79–0.97)	0.13	0.74	0.90

Best-performing classifier per predictor block (logistic regression or random forest). Lower Brier scores indicate better probabilistic prediction accuracy. Calibration slope values closer to 1 indicate improved agreement between predicted and observed risk. LOCO = leave-one-center-out pooled validation. Bootstrap 95% confidence intervals are shown for AUC estimates.

## Data Availability

De-identified analytic dataset and complete analysis code are available from the corresponding author upon reasonable request.
